# BMP/Smad Pathway Is Involved in Lithium Carbonate-Induced Neural-Tube Defects in Mice and Neural Stem Cells

**DOI:** 10.3390/ijms232314831

**Published:** 2022-11-27

**Authors:** Aiyun Yang, Shen Li, Yan Zhang, Xiuwei Wang, Zhen Guan, Zhiqiang Zhu, Yingchao Liang, Lijiao Zhao, Jianhua Wang

**Affiliations:** 1 Translational Medicine Laboratory, Beijing Municipal Key Laboratory of Child Development and Nutriomics, Capital Institute of Pediatrics, Beijing 100020, China; 2 Beijing Key Laboratory of Environmental & Viral Oncology, College of Life Science & Bioengineering, Beijing University of Technology, Beijing 100124, China

**Keywords:** inositol, BMP signaling, neural-tube defects, neural stem cell, proliferation

## Abstract

Neural-tube defects (NTDs) are one type of the most serious birth defects. Studies have shown that inositol deficiency is closely related to the occurrence of NTDs. Bone morphogenetic protein (BMP)-mediated Smad signaling pathways have been implicated in neurogenesis and neural-tube closure. However, the role of the BMP/Smad pathway in inositol-deficiency-induced NTDs remains unclear. Inositol-deficiency models in C57 mice and mouse neural stem cells (mNSCs) were induced with Li_2_CO_3_ treatment or inositol withdrawal. The role of the BMP/Smad pathway in the regulation of cell proliferation and the development of NTDs was determined utilizing qRT-PCR, HE staining, Western blot, immunostaining, MTT assay, EdU staining, and flow cytometry. The intraperitoneal injection of Li_2_CO_3_ at Embryonic Day 7.5 induced the occurrence of NTDs. The mRNA levels of *Bmp2*, *Bmp4*, *Smad1*, *Smad5*, *Smad8* and *Runx2*, the phosphorylation of Smad1/5/8, and the nuclear translocation of Runx2 were significantly increased in NTD embryonic brain tissues and mNSCs exposed to Li_2_CO_3_ or an inositol-free medium, which were suppressed by BMP receptor selective inhibitor LDN-193189. The Li_2_CO_3_-induced phosphorylation of Smad1/5/8 was inhibited by inositol supplementation. Cell proliferation was significantly promoted by Li_2_CO_3_ exposure or the absence of inositol in mNSCs, which was reversed by LDN-193189. These results suggest that the activation of the BMP/Smad signaling pathway might play an important role in the development of NTDs induced by maternal Li_2_CO_3_ exposure via inositol deficiency.

## 1. Introduction

Neural-tube defects (NTDs) are the second most common congenital malformations in humans, characterized by the impaired development of the central nervous system (CNS) [1]. The global incidence is about 1.86‰ [2,3]. The main manifestations of fetal neural-tube malformation include anencephaly, encephalocele, encephalomeningocele, and spina bifida [2]. Vertebrate neurulation is a complex morphogenetic process that requires the coordination of many cellular and molecular events. The neural tube closes from an open neural plate (NP). The NP is initially induced to differentiate, undergoes bending to create the neural folds that are elevated towards the dorsal midline, and the neural-fold tips lastly fuse to complete the neural tube. The failure of the dynamic morphological changes of neurulation leads to perturbations in neural-tube closure, generating NTDs. Genetic and environmental risk factors in the background of NTDs have been identified and extensively reported. Maternal folic acid deficiency is an important environmental factor in the cause of NTDs. Folic acid, a water-soluble vitamin B, is required for purine and pyrimidine synthesis, DNA repair, and methylation reactions [4]. During embryonic neural-tube development, rapidly dividing cells require large amounts of nucleotide synthesis to facilitate DNA replication. Folic acid deficiency impairs thymidylate synthesis [5] and the methylation process [6], which affect the normal cell proliferation and differentiation, resulting in the disruption of the development of neural-tube closure. Folic acid administration in early pregnancy is effective in preventing the occurrence of NTDs [1,7]. However, folic acid supplementation cannot completely prevent the incidence of NTDs, as more than 30% of NTDs are resistant to folic acid supplementation [8,9].

Inositol, another water-soluble vitamin B, has a different metabolic process than that of folic acid [5,10]. Perinatal inositol supplementation could effectively reduce the incidence of NTDs associated with folic acid resistance [11]. Population-based studies showed that the serum inositol level was significantly decreased in pregnant women with NTDs; the plasma inositol level was 7% lower in children with spina bifida than that in healthy children [12,13]. The polymorphisms of inositol-metabolism-related genes, such as inositol transporter (*SLC5A11*, encodes SC5AB), inositol synthase (*ISYNA1*, encodes inositol monophosphatase, IMPase) [10], and 1,3,4-triphosphate inositol 5/6-kinase (*ITPK1*, encodes inositol-tetrakisphosphate 1-kinase) [14], were significantly associated with NTDs. These data suggest that inositol deficiency is an important nutritional risk factor for the development of NTDs.

Lithium carbonate (Li_2_CO_3_) is a potent inhibitor of IMPase that dephosphorylates inositol monophosphate into free inositol in the phosphatidylinositol (PI) system. Li_2_CO_3_ thereby depletes the endogenous source of inositol in cells and prevents the production of free inositol [15]. We reported previously that maternal exposure to Li_2_CO_3_ induced the occurrence of NTDs in mice with a decreased plasma inositol level through inhibiting IMPase activity, suggesting that Li_2_CO_3_ treatment caused inositol deficiency in mice [16]. This provides us with an alternative animal model to study the mechanisms of inositol-deficiency-induced NTDs. Li_2_CO_3_ is widely used to treat mental illness and stabilize mood changes in pregnant women [17]. The therapeutic window of Li_2_CO_3_ is relatively narrow, and the normal range of lithium in human serum is from 0.6 to 1.2 mM [18]. Lithium toxicity might occur when the concentration of lithium in the blood rises to more than 1.4 mM [19]. Therefore, taking Li_2_CO_3_ for a long time requires the regular monitoring of blood lithium concentration. Since lithium can directly cross the placenta [20,21], its teratogenic effects on various systems were reported in pregnant women and animals, such as the nervous and cardiovascular systems [22,23,24,25]. Treatment with myo-inositol completely reversed the effects of lithium on cardiovascular malformation in a time-dependent manner [25]. This could be of particular importance in providing an alternative for preventing maternal Li_2_CO_3_-exposure-induced NTDs and unresponsive NTDs to folic acid.

Bone morphogenetic proteins (BMPs) belong to the TGF-β superfamily, and the BMP family consists of numerous ligands (such as BMP2/4, BMP5/6/7/8, BMP9/10), Type 1 and 2 receptors, and Smads (Smad1, Smad5, and Smad8/9). BMP proteins form dimers, and bind to and activate BMP Type 2 and 1 receptors, which then recruit and phosphorylate Smad1/5/8 (p-Smad) [26]. The activated p-Smads form complexes with Smad4 and then transport it into the nucleus to regulate target gene expression. The activities of BMPs and downstream effectors are dynamically regulated during gastrulation and dorsoventral patterning within the neural tube, and in adult brain homeostasis and functions [27]. The canonical BMP pathway (Smad1/5/8-dependent) plays important roles in neural stem cell fate decisions during neurogenesis [28,29]. BMP2 was required for cephalic neural-tube closure in mice [30]. BMP regulates the closure of the neural tube by adjusting the tight junctions [31] and hinge points [32]. Studies showed that lithium was involved in regulation of the expressions of BMP2 and BMP4 in murine mesenchymal stem cells and preosteoblasts [33,34]. On the basis of these data, we propose that the BMP/Smad signaling pathway is involved in Li_2_CO_3_-induced NTDs via inositol deficiency. In this study, we established Li_2_CO_3_-induced inositol deficiency NTD models in mice and mouse neural stem cells (mNSCs) to explore the role of the BMP/Smad pathway in the regulation of the development of NTDs.

## 2. Results

### 2.1. Maternal Exposure to Li_2_CO_3_ Induced the Development of NTDs in Mice

**Figure 1:**
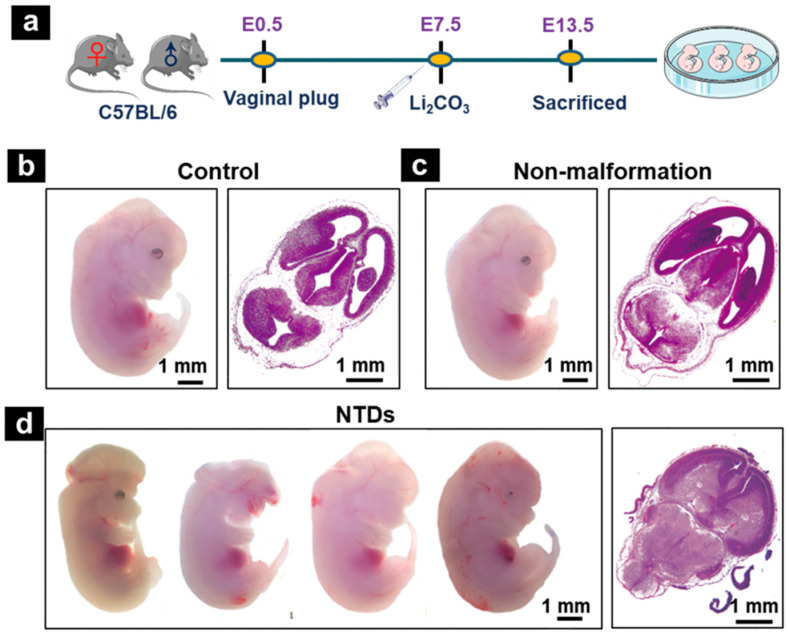
Neural-tube defects (NTDs) mouse model induced by Li_2_CO_3_. (**a**) Schematic diagram of the study design. Li_2_CO_3_ was injected intraperitoneally at E 7.5, and embryos were isolated and collected at E 13.5. (**b**–**d**) Embryonic morphology at E 13.5 and representative HE staining viewed under microscope. (**b**) Control group; (**c**) nonmalformation group; (**d**) NTDs group. Scale bar = 1 mm.

In this study, Li_2_CO_3_ was injected intraperitoneally at E 7.5 (before neural-tube closure) in pregnant mice (Figure 1a). The embryos were isolated and observed under a stereomicroscope at E 13.5. Embryos in the control group were well-developed. In the Li_2_CO_3_ group, the average incidence of NTDs was 30.0% (Table S1). The embryos in the control group had a plump and smooth appearance, and complete tissue structure characteristics (Figure 1b). The phenotype in the Li_2_CO_3_-induced NTD group showed abnormal development and obvious NTDs, manifested as anophthalmia, craniofacial malformation, and growth retardation (Figure 1d). The HE staining of embryonic brain tissue sections under a light microscope showed that the neural tube in the control group was completely closed, the hindbrain was well-developed, and the mesenchymal cells in the surrounding ventricles were evenly distributed and dense (Figure 1b). The fourth ventricle and telencephalon were dysplastic in the mouse models of NTDs, and the neuroepithelial cells were disorganized (Figure 1d). Although no obvious phenotypic abnormalities were found under the dissecting microscope, in the Li_2_CO_3_-treated nonmalformation group, HE staining showed a narrower ventricular cavity of the hindbrain and midbrain, compared to that of the control group (Figure 1c). These results are consistent with our previous study [16,35]. The process of neural-tube closure was disrupted by maternal Li_2_CO_3_ exposure during embryonic development.

### 2.2. Increased Expression and Activation of the BMP/Smad Signaling Pathway in Embryonic Brain Tissues with NTDs

**Figure 2:**
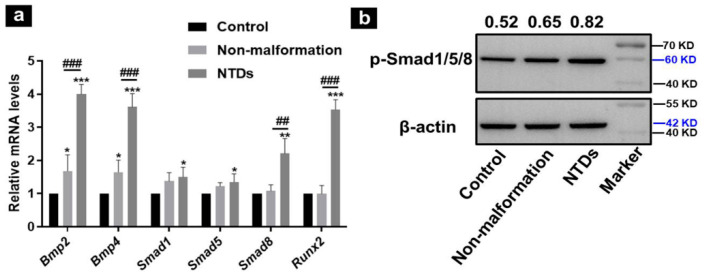
Activation of the BMP/Smad signaling pathway in embryonic brain tissues at E 13.5. (**a**) mRNA levels of *Bmp2, Bmp4, Smad1/5/8 and Runx2* were analyzed with qRT-PCR. (**b**) The phosphorylation of Smad1/5/8 was determined with Western blotting. β-actin was used as loading control. Data are shown as mean ± SD (n = 3; * *p* < 0.05, ** *p* < 0.01 and *** *p* < 0.001 vs. control group; ## *p* < 0.01, ### *p* < 0.001 vs. the nonmalformation group).

To determine the role of the BMP/Smad pathway in the development of NTDs, embryonic brain tissues were collected, and the mRNA expression of key genes in the BMP/Smad pathway was determined with qRT-PCR. The results show that the mRNA levels of *Bmp2*, *Bmp4*, *Smad1*, *Smad5*, *Smad8*, and *Runx2* were significantly increased in the NTDs group compared to those of the normal control (Figure 2a). A significantly increased gene expression of *Bmp2*, *Bmp4*, *Smad8*, and *Runx2* was observed in the NTDs group compared to the Li_2_CO_3_-treated nonmalformation group. The gene expression of *Bmp2* and *Bmp4* was also increased in the nonmalformation group compared to that in the normal control, suggesting that the maternal exposure to Li_2_CO_3_ upregulated the gene expressions of BMP/Smad signaling molecules even in embryos without a significant developed malformation. Next, we examined the activation of the BMP/Smad pathway with Western blotting. As shown in Figure 2b, the phosphorylation of Smad1/5/8 was significantly increased in the NTDs group compared to that in the control and nonmalformation groups. These results suggest that Li_2_CO_3_ treatment promoted the activation of the BMP/Smad signaling pathway.

### 2.3. Role of Inositol in Li_2_CO_3_ Promoted Activation of BMP/Smad Signaling Pathway in mNSCs

**Figure 3:**
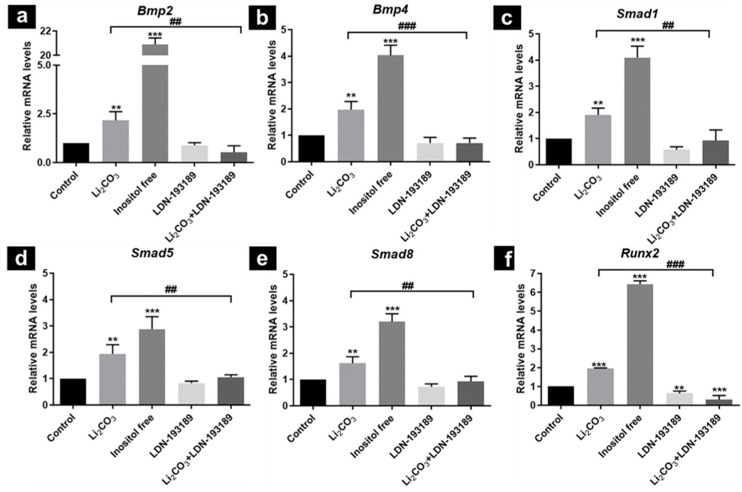
Gene expressions of BMP signaling molecules in mNSCs. Cultured mNSCs were exposed to Li_2_CO_3_ (1.5 mM), or inositol-free medium, treated with or without LDN-193189 (1 μM). Gene expressions of (**a**) *Bmp2*, (**b**) *Bmp4*, (**c**) *Smad1*, (**d**) *Smad5*, (**e**) *Smad8*, and (**f**) *Runx2* were determined. Data are shown as mean ± SD (n = 5); ** *p* < 0.01 and *** *p* < 0.001 vs. control group; ## *p* < 0.01 and ### *p* < 0.001 comparing the Li_2_CO_3_ and Li_2_CO_3_ + LDN-193189 groups.

Li_2_CO_3_ inhibits the activity of IMPase, a key enzyme for inositol synthesis in vivo [36,37]. We previously reported that maternal Li_2_CO_3_ exposure induced inositol deficiency play a critical role in the development of NTDs [16,35]. To determine the role of inositol in the Li_2_CO_3_-regulated BMP/Smad pathway, mNSCs were cultured in a normal MEM medium (inositol concentration was 0.01 mM) or inositol-free MEM, treated with or without Li_2_CO_3_ or LDN-193189 (BMP receptor selective inhibitor). As shown in Figure 3, the mRNA levels of *Bmp2*, *Bmp4*, *Smad1*, *Smad5*, *Smad8* and *Runx2* significantly increased in cells exposed to Li_2_CO_3_. These results are consistent with the in vivo data that we observed in NTD embryonic brain tissues. The effect of the inositol-free medium on the gene expression of *Bmp2*, *Bmp4*, *Smad1*, *Smad5*, *Smad8* and *Runx2* was more obvious compared to the Li_2_CO_3_ treatment. The Li_2_CO_3_-induced upregulation of *Bmp2*, *Bmp4*, *Smad1*, *Smad5*, *Smad8* and *Runx2* was suppressed by LDN-193189. Interestingly, LDN-193189 treatment alone significantly reduced the mRNA levels of the target gene *Runx2*, but no significant changes were observed in other key genes.

**Figure 4:**
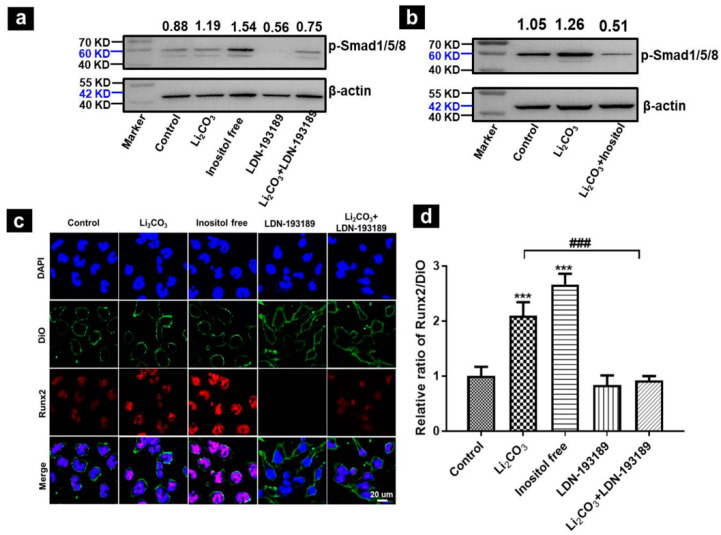
Activation of Smad signaling pathway in mNSCs. (**a**) Western blot of p-Smad1/5/8. (**b**) Expression of p-Smad1/5/8 after inositol (10 mM) supplementation. (**c**) Confocal images of mNSCs labeled with Runx2 antibody (red), Dio (green), and cell nucleus (DAPI, blue). The scale bar is 20 µm for all. (**d**) Quantification result of Runx2 in (**c**). Li_2_CO_3_: 1.5 mM, LDN-193189: 1 μM. Data are shown as mean ± SD. (n = 3; *** *p* < 0.001 vs. control group; ### *p* < 0.001, comparison between Li_2_CO_3_ and Li_2_CO_3_ + LDN-193189 groups).

Results from Western blotting show a significantly increased level of p-Smad1/5/8 in Li_2_CO_3_-treated mNSCs and in cells under inositol-free condition. The Li_2_CO_3_-induced phosphorylation of Smad1/5/8 was inhibited by LDN-193189 (Figure 4a). Moreover, the Li_2_CO_3_-induced phosphorylation of Smad1/5/8 was inhibited by 10 mM inositol supplementation, indicating that inositol had an inhibitory effect on BMP/Smad signaling, and that the decreased inositol level was associated with the Li_2_CO_3_-induced activation of the BMP/Smad signaling pathway (Figure 4b).

To determine whether the target protein Runx2 had been activated, mNSCs were multiple-labeled and subjected to a laser confocal microscope. The results show that the Runx2 protein was localized in the nucleus, and the fluorescence intensity of Runx2 increased in the Li_2_CO_3_ or inositol-free group, which was inhibited by LDN-193189 (Figure 4c,d). These data suggest that Li_2_CO_3_-induced inositol deficiency was involved in the upregulated expression of BMPs, resulting in the activation of the Smad1/5/8 pathway and downstream *Runx2*.

### 2.4. Role of the BMP/Smad Signaling Pathway in the Regulation of Cell Proliferation in mNSCs

**Figure 5:**
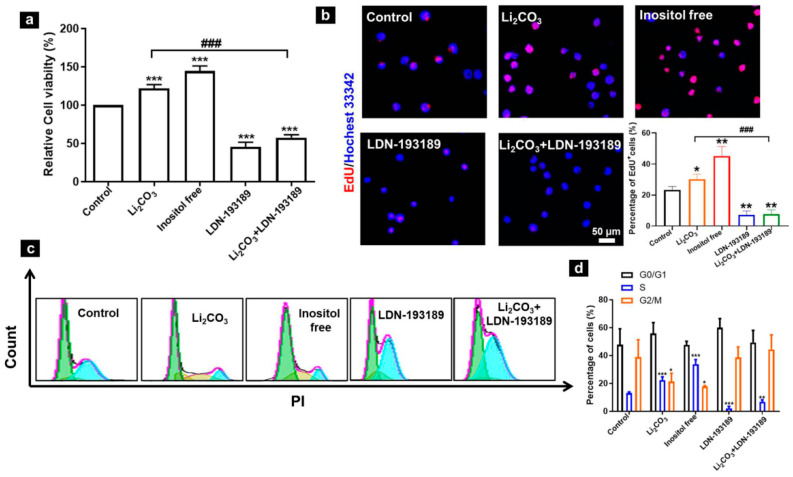
Role of BMP/Smad signaling in the regulation of cell proliferation in mNSCs. (**a**) MTT assay (n = 6). (**b**) EdU incorporation assay showing the percentages of EdU-positive cells (red). Nuclei were visualized with Hoechst 33342 (blue) and analyzed with fluorescent microscopy. Scale bar = 50 μm. (**c**) Representative cell-cycle analysis performed with flow cytometry. (**d**) Percentage of cells in each phase. Data represented as mean ± SD (n = 3; * *p* < 0.05, ** *p* < 0.01 and *** *p* < 0.001 vs. control group; ### *p* < 0.001, comparison between the Li_2_CO_3_ and Li_2_CO_3_ + LDN-193189 groups).

Our previous study showed that maternal Li_2_CO_3_ exposure promoted cell proliferation in neuroepithelial cells [16]. To determine whether the BMP/Smad signaling pathway was involved in inositol-deficiency-induced cell proliferation, an MTT assay was performed in mNSCs. There was significantly increased cell viability (proliferation) in mNSCs treated with Li_2_CO_3_ or an inositol-free medium, which was inhibited by LDN-193189. Cell viability was reduced to 45.7% and 57.6% in mNSCs treated with LDN-193189 alone or Li_2_CO_3_ plus LDN-193189, respectively (Figure 5a). An EdU assay further confirmed our finding. The percentage of EdU-positive cells increased significantly under the inositol-free condition or treatment with Li_2_CO_3_, which was suppressed by LDN-193189 (Figure 5b). Simultaneously, a substantial increase in S-phase cells was detected in cells treated with Li_2_CO_3_ or the inositol-free medium (Figure 5c,d); the percentage of S-phase cells increased from 13.1% (control group) to 22.4% (Li_2_CO_3_ group) and 33.8% (inositol-free group). After LDN-193189 treatment, the proportion of S-phase cells decreased to 2.1% (LDN-193189 group) and 6.8% (Li_2_CO_3_ + LDN-193189 group). Therefore, the Li_2_CO_3_-induced activation of the BMP/Smad signaling pathway contributed to increased cell proliferation in mNSCs via inositol deficiency.

## 3. Discussion

Inositol deficiency is a risk factor in the occurrence of NTDs. However, no appropriate inositol-deficiency animal model had been established till now. Li_2_CO_3_ is a potent inhibitor of IMPase, thereby preventing the production of free inositol, resulting in an inositol-deficiency condition. Thus, in this study, we established a Li_2_CO_3_-induced NTDs mouse model to study the mechanism of inositol deficiency in the development of NTDs. Using an in vivo Li_2_CO_3_-induced NTD mouse model and in vitro cultured mNSCs, we demonstrated that inositol deficiency is an important contributor in Li_2_CO_3_-induced NTDs, and the activation of BMP/Smad signaling is involved in inositol deficiency, promoting neural stem cell proliferation. These results suggest that BMP/Smad signaling might play an important role in Li_2_CO_3_-induced NTDs.

Inositol deficiency is closely related to abnormal embryonic neurodevelopment [38,39,40], but the involved signaling mechanism has not been elucidated. Studies showed that BMP is a key factor in ectodermal epidermal formation and the inhibition of ectodermal neural-tube closure [41,42]. It activates downstream effectors Smad-1/5/8 and target gene *Runx2* to regulate normal neural-tube development [43]. The expression of BMP/Smad signaling molecules is increased during abnormal brain development [44,45,46]. In this study, we observed an increased gene expression of *Bmp2*, *Bmp4*, *Smad1*, *Smad5*, *Smad8*, and *Runx2*, the phosphorylation of Smad1/5/8, and the activation of Runx2 in Li_2_CO_3_-induced NTD mouse embryonic neural tissues and in mNSCs exposed to Li_2_CO_3_ or an inositol- free medium, and that inositol-free condition had more evident effects. Although we did not measure the inositol levels in this study, our previous data demonstrated that the plasma levels of inositol in maternal Li_2_CO_3_-exposed mice and in embryonic brain tissues were significantly decreased [16]. These results suggested that Li_2_CO_3_ exposure induced an inositol deficiency condition, which might be the major contributor in the development of NTDs. In the present study, we found that inositol supplementation significantly inhibited the Li_2_CO_3_-induced phosphorylation of Smad1/5/8. These results indicate that inositol deficiency promotes the activation of the BMP/Smad pathway, and that inositol was required in maintaining the precise regulation of BMP/Smad signaling during neural-tube closure. To understand how Li_2_CO_3_ regulates the activation of the BMP pathway, LDN-193189 was utilized. LDN-193189 primarily inhibits BMP Type I receptors (activin receptor-like kinase 3, ALK3) and ALK6, with some inhibition of ALK1 and ALK2, as demonstrated in C2C12 osteoblast and chondroblast cell lines [47]. The mechanism of inhibition involves the competitive binding of the compound to the kinase domain of Type I subunits, preventing phosphorylation of downstream Smad molecules and restricting the signaling cascade [47]. LDN-193189 could effectively block the phosphorylation of Smad 1/5/8 [47], and inhibit the expression of target gene *Runx2* [48]. We observed that Li_2_CO_3_ and inositol deficiency induced the gene expression of *Bmp2*, *Bmp4*, *Smad1*, *Smad5*, *Smad8* and *Runx2*, the phosphorylation of Smad1/5/8, and the nuclear translocation of Runx2 in vivo and in vitro. LDN-193189 treatment significantly inhibited the upregulated gene expression and activation of BMP/Smad signaling molecules in mNSCs. These results suggest that Li_2_CO_3_ either directly functioned as an extracellular activator or indirectly acted through eliminating the inhibitory effect of inositol by reducing inositol level to promote the expression/activation of the BMP/Smad signaling pathway.

**Figure 6:**
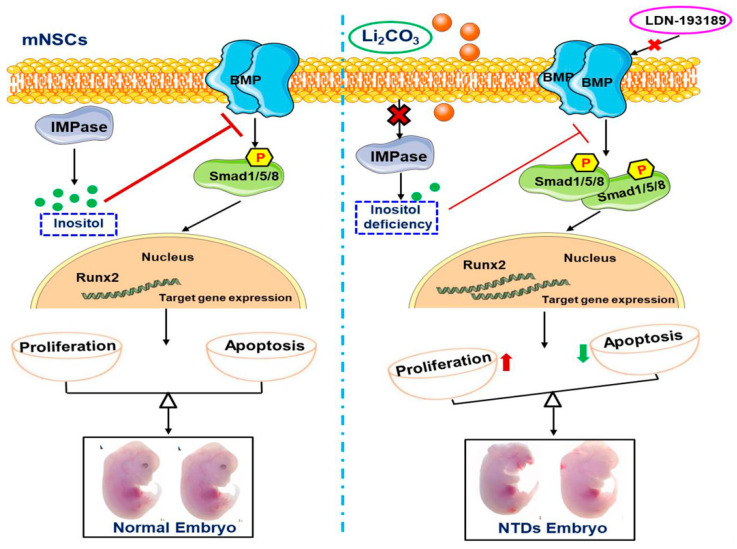
Schematic of mechanisms on Li_2_CO_3_-induced neural-tube defects. Li_2_CO_3_ inhibited IMPase and caused inositol deficiency in neural stem cells. Li_2_CO_3_ stimulated the gene expressions of BMPs and activated the downstream Smad1/5/8-mediated signaling pathway by eliminating the inhibitory effects of inositol, which was reversed by LDN-193189. The activation of the BMP/Smad signaling pathway promoted cell proliferation, and disrupted the balance between cell proliferation and apoptosis, which was likely associated with the development of NTDs.

During the development of the neural tube, dynamically balanced cell proliferation and apoptosis are required to ensure normal neural-tube closure. Once the balance of proliferation and apoptosis is broken, NTDs are likely to happen [49,50]. Mutations in genes regulating cell proliferation and apoptosis led to the development of NTDs in mouse embryos [51]. We observed increased cell proliferation in mNSCs exposed to Li_2_CO_3_ or cultured with inositol-free medium, and the number of cells in the S phase also increased significantly, suggesting that Li_2_CO_3_ exposure or inositol deficiency promoted cell proliferation. LDN-193189 reversed the excessive proliferation of mNSCs, indicating that the activated BMP/Smad signaling pathway was involved in Li_2_CO_3_-induced cell proliferation. LDN-193189 could attenuate the cell proliferation in periodontal ligament stem cells and prostate cancer cells [52,53]. Although cell apoptosis was not analyzed in this study, Li_2_CO_3_ has antiapoptotic effects [54,55,56,57], and our previously study also demonstrated that the expression of cleaved caspase-3 and P53 was decreased in a Li_2_CO_3_-induced NTDs mouse model [16], suggesting that the cell apoptotic process was inhibited following Li_2_CO_3_ exposure. It was speculated that Li_2_CO_3_ and/or inositol deficiency might cause excessive proliferation and reduced apoptosis via the BMP/Smad signaling pathway. The imbalanced cell proliferation and apoptosis during neural-tube development would ultimately lead to the occurrence of NTDs (Figure 6).

The present study opens a new direction in understanding the role of the BMP/Smad signaling pathway in the development of NTDs. BMP molecules may serve as potential markers for NTDs, such as alpha-fetoprotein (AFP) and proprotein convertase subtilisin/kexin type 9 (PCSK9) [58]. In addition, there are some limitations in our study. Two or more cell lines, such as mouse embryonic stem cells (mESCs) and human neural stem cells (hNSCs), can be used to validate the experimental results. LDN-193189 treatment in the animal models would further confirm the role of the BMP/Smad signaling pathway in Li_2_CO_3_ -induced NTDs.

## 4. Materials and Methods

### 4.1. NTDs Mouse Model Establishment

The NTDs mouse model was established according to a previously reported procedure [16]. Animal experiments were conducted in accordance with the National Institutes of Health Animal Care Standards, and approved by the Ethics Committee of the Capital Institute Pediatrics (DWLL2021013). Briefly, C57BL/6 mice (7–9 weeks old, weighing 19–22 g) were maintained at the experimental animal center under specific pathogen-free conditions. Laboratory utensils, feeds, litter, cages, and other items were autoclaved before being taken into the laboratory. After being acclimatized to the environment for 1 week, female mice were mated with males overnight (1:1, from 6 p.m. to 8 a.m.). The vaginal plugs were detected the next morning, which was considered to be Embryonic Day 0.5 (E 0.5) when present. Pregnant mice were randomly divided into 2 groups with 8 mice in each group. The control group was treated with 0.9% saline, while the experimental group was treated with 350 mg/kg Li_2_CO_3_ (Sigma-Aldrich, ST Louis, MO, USA) by intraperitoneal injection on E 7.5.

### 4.2. Embryos Examination and Sample Collection

Pregnant mice were euthanized at E 13.5. The embryos were explanted into Hank’s balanced salt solution (Life Technologies Inc., Burlington, ON, Canada) for morphological and histological studies. Embryos were examined for external malformations under a dissecting microscope (SZ2-ILST, Olympus, Tokyo, Japan). Embryos from Li_2_CO_3_ treated group were divided into non-malformation group (no visible malformation) and NTDs group. Embryos collected from the saline group served as controls. The embryonic brain tissues collected from the control, nonmalformation, and NTDs groups were cryopreserved at −80 °C for subsequent experiments.

### 4.3. Hematoxylin and Eosin (HE) Staining

In order to observe the structure of the brain tissue and the arrangement of neuroepithelial cells, HE staining was performed. Embryos fixed in 4% paraformaldehyde (Aladdin, Shanghai, China) were embedded in paraffin and sliced. The sections were incubated with hematoxylin dye solution for 5 min. After turning blue, sections were washed with tap water, and an eosin dye solution was added for 3 min. The stained sections were treated with 75% and 85% alcohol for gradient dehydration for 2 min. After incubation with xylene for 1 min, sections were sealed, observed, and photographed using an optical microscope (BX53, Olympus, Japan).

### 4.4. Cell Culture

Mouse neural stem cells (NE-4C) were purchased from the Stem Cell Bank of the Chinese Academy of Sciences. Before cell culture, culture flaps/plates were coated with 1 μg/cm^2^ poly-D-lysine (PDL, Millipore, MA, USA) to help cells in adhering. Cells were cultured in an MEM medium (Gibco, Grand Island, USA) containing 10% FBS (Gibco), 1% nonessential amino acids (Gibco), 1% glutamine (Gibco), and 1% penicillin/streptomycin (Gibco). The concentration of inositol in the MEM medium was 0.01 mM. Cells were passed on in a ratio of 1:10 when they grew to the logarithmic stage. After starvation, cells were subjected to different treatments: Li_2_CO_3_ (1.5 mM), inositol-free (inositol-free MEM medium), LDN-193189 (BMP receptor selective inhibitor, 1 μM, MCE, USA), Li_2_CO_3_ + LDN-193189 (1.5 mM Li_2_CO_3_ +1 μM LDN-193189) or Li_2_CO_3_ + inositol supplementation (1.5 mM Li_2_CO_3_ + 10 mM inositol). Cells were maintained at 37 °C in a humidified atmosphere with 5% CO_2_.

### 4.5. MTT Assay

Cells were seeded into 96-well plates at a density of 1.5 × 10^4^ cells/well and cultured for 24 h. Cells were subjected to different treatments as described above at 37 °C for 24 h, followed by washing with PBS and incubation with a fresh medium containing 10 uL 5 mg/mL MTT (Solarbio, Beijing, China) for 4 h. After discarding the liquid, 150 µL DMSO was added into each well and incubated for 10 min. The absorbance of each well was measured at 450 nm using the Synergy H1 hybrid multifunction microplate reader (BioTek Instruments, Winooski, VT, USA). The measurements were carried out in six parallel lines, and the relative cell viability was expressed as a percentage of the control.

### 4.6. EdU Assay

EdU assay was performed to determine the proliferation of mNSCs, using the EdU Cell Proliferation Kit, according to the manufacturer’s instructions (Shanghai Epizyme Biomedical Technology Co., Ltd., Shanghai, China). After subjecting to different treatment, mNSCs were incubated with 50 nM EdU for 2 h. The proliferating cells were fixed with 4% paraformaldehyde. Cell nuclei were stained with Hoechst 33342. The proportion of cells that incorporated EdU was visualized and determined under a laser scanning confocal microscope (SP8, Leica, Wetzlar, Germany). Three equally sized fields were randomly chosen, and the positive cells was counted. Data are presented as mean ± SD.

### 4.7. Cell-Cycle Assay

Cells were seeded and cultured into 6-well plates at a density of 1 × 10^6^ cells per dish. After 24 h of serum starvation, cells were subjected to different treatments as described above for 24 h. The cells were harvested, washed twice with PBS, and the cell precipitate was resuspended with 100 μL PBS. Then, 1 mL of precooled 75% ethanol was slowly added to each sample and fixed overnight at 4 °C. After washing, fixation, and treatment with 100 μL RNnase (20 μg/mL, Tiangen, Beijing, China), cells were stained with PI (50 μg/mL, Sigma, St. Louis, MO, USA) and filtered through a 35 µm strainer cap before being subjected to flow cytometry (Beckman, CA, USA). FlowJo software was used to analyze the cell-cycle distribution.

### 4.8. Quantitative RT-PCR (qRT-PCR)

Total RNA were extracted from the serum-starved mNSCs and embryonic brain tissues (E 13.5, n = 3) using the RNeasy R Micro Kit (Qiagen, Germany). The quantity of RNA was analyzed with a Nanodrop 2000 spectrophotometer (Thermo Fisher Scientific, Waltham, MA, USA) and then reversely transcribed into cDNA system. qRT-PCR was performed using an SYBR probe (Takara Bio, Kusatsu, Japan), and fluorescence was detected with a CFX96 Connect Real-Time PCR Detection System (Biorad, Hercules, CA, USA). *BMP2/4*, *Smad1/5/8* and *Runx2* (runt-related transcription factor 2) were the key genes of the BMP/Smad signaling pathway. The primers were purchased from Sangon Biotech (Shanghai, China), and the sequences are listed in Table 1. The reaction was carried out in a 25 μL system. After the reaction, the relative expression of the target gene was calculated according to the average value of threshold cycle (Ct value) of the target gene and the housekeeping gene, which were expressed as 2^−△△Ct^.

### 4.9. Western Blot

Total protein was extracted from embryonic brain tissues and cultured mNSCs. The protein concentrations were determined using the PierceTM BCA Protein Assay Kit (Thermo Fisher Scientific, Waltham, MA, USA), and the lysates were then mixed fully with a 5× sample buffer. Equal protein amounts (30 µg) were subjected to SDS-PAGE and electrotransferred onto a PVDF membrane (Merck Millipore, Darmstadt, Germany). The membrane was blocked with 5% nonfat milk in Tris-buffered saline containing 0.1% Tween-20 (TBST) for 1 h at room temperature. The protein expression of p-Smad1/5/8 (Cell Signaling Technology, Boston, MA, USA, dilution rate, 1:1000) was detected with Western blotting. The membrane was stripped for an hour after the p-Smad1/5/8 had been photographed, and then blocked to detect the loading control (β-actin, Cell Signaling Technology, Boston, MA, USA, dilution rate, 1:1000), washed with TBST for three times, and imaged with a gel imaging system (Tanon Science & Technology, Shanghai, China). The individual band in the Western blot was semiquantified with ImageJ software.

### 4.10. Immunofluorescence

To determine the expression of Runx2, a transcription factor downstream of BMP/Smad signaling, mNSCs were seeded and cultured into 24-well plates (preloaded with cell climbing glass slices) at a density of 4 × 10^4^ cells per well. Cells were subjected to different treatments as described above at 37 °C for 24 h, followed by washing with PBS. Cells were fixed with 4% polyformaldehyde (Beijing Chemical Reagent Company, Beijing, China) at 37 °C for 30 min and permeabilized using 0.3% Triton X-100 (Beijing Chemical Reagent Company) for 15 min. After rinsing with PBS and incubating with 2% albumin from bovine serum (BSA, Sigma, USA) for 1 h, anti-Runx2 antibody (Cell Signaling Technology, Boston, MA, USA, dilution rate, 1:600) and 594 conjugated goat antirabbit IgG (H + L) (Cell Signaling Technology, Boston, MA, USA, dilution rate, 1:600) were used for fluorescence staining. After incubation with DiO (1:500, cell membrane dye) for 10 min, slides were mounted with a DAPI-containing mounting medium (Zhongshan Goldenbridge biotechnology Co., Beijing, China). The microphotographs were taken with a confocal microscope (SP8, Leica, Germany), and the data analyzed with ImageJ. Relevant image acquisition parameters: UV: 351, 364 nm; blue light: 458, 476 nm; green light: 488, 568 nm; red light: 647; size: 512 × 512; gain: about 500.

### 4.11. Statistical Analysis

All the quantitative data are expressed as mean ± standard deviation. One-way analysis of variance was used to assess the statistical significance of the experimental results. Statistical analysis was performed using SPSS software (SPSS 20.0). ImageJ software was used for the quantification of confocal images and Western blot bands. FlowJo and GraphPad Prism 8.0 software was utilized to analyze the cell-cycle distribution and data, respectively. Results were considered to be statistically significant at *p* < 0.05.

## 5. Conclusions

In conclusion, the activation of the BMP/Smad signaling pathway might play an important role in the development of NTDs induced by maternal exposure to Li_2_CO_3_ via inositol deficiency. This study provided new ideas for further elucidating the molecular mechanism of inositol deficiency in embryonic neurodevelopmental abnormalities, which might help in the early diagnosis and prevention of neurodevelopmental defects.

## Figures and Tables

**Table 1:** PCR primers of the key genes in the BMP/Smad pathway.

Gene	Forward Primer	Reverse Primer
*Bmp2*	5′-TTGGACACCAGGTTAGTGAATCA-3′	5′-TCTCCTCTAAATGGGCCACTT-3′
*Bmp4*	5′-TTGATACCTGAGACCGGGAAG-3′	5′-ACATCTGTAGAAGTGTCGCCTC-3′
*Smad1*	5′-GACGCTTTGGTGAAGAAACTGA-3′	5′-GGGAGCGAGGAATGGTGAC-3′
*Smad5*	5′-CCAGCCGTGAAGCGATTGT-3′	5′-CTCCTCCATAGCACCCTTCT-3′
*Smad8*	5′-CGGGTCAGCCTAGCAAGTG-3′	5′-GAGCCGAACGGGAACTCAC-3′
*Runx2*	5′-CCGGTCTCCTTCCAGGAT-3′	5′-GGGAACTGCTGTGGCTTC-3′
*Gapdh*	5′-GGTCACCAGGGCTGCTTTTA-3′	5′-GAGGGATCTCGCTCCTGGA-3′

## Data Availability

The materials described in the manuscript, including all relevant raw data are freely available from the corresponding author to any researcher wishing to use them for noncommercial purposes, without breaching participant confidentiality.

## Supplementary Materials

The following supporting information can be downloaded at: https://www.mdpi.com/article/10.3390/ijms232314831/s1.
