# Supplementary material for: BMP/Smad Pathway Is Involved in Lithium Carbonate-Induced Neural-Tube Defects in Mice and Neural Stem Cells
Source: Int J Mol Sci. 2022 Nov 27;23(23):14831. doi: 10.3390/ijms232314831 (PMC9735442; doi:10.3390/ijms232314831)
Supplement: Supplementary file 1 [file ijms-23-14831-s001.zip › ijms-1959208-supplementary.pdf]

International Journal of *Molecular Sciences*

MDPI

Supplementary materials for

BMP/Smad Pathway Is Involved in Lithium Carbonate-Induced Neural Tube Defects in Mice and Neural Stem Cells

Aiyun Yang [1,†], Shen Li [1,†], Yan Zhang [2], Xiuwei Wang [1], Zhen Guan [1], Zhiqiang Zhu [1], Yingchao Liang [1], Lijiao Zhao [2] and Jianhua Wang [1,*]

1 Translational Medicine Laboratory, Beijing Municipal Key Laboratory of Child Development and Nutriomics, Capital Institute of Pediatrics, Beijing 100020, China
2 Beijing Key Laboratory of Environmental & Viral Oncology, College of Life Science & Bioengineering, Beijing University of Technology, 100124, Beijing, China
* Correspondence: fywjh@163.com

Table S1. Murine embryonic development by Li_2CO_3 treatment.

Batch number	Group	Litters *n*	Embryos *n*	Normal *n* (%)	Resorption *n* (%)	NTDs *n* (%)	Other malformations *n* (%)
1	Control	4	35	34 (97.1)	1 (2.9)	0 (0)	0 (0)
	Li_2CO_3	4	30	11 (36.7)	6 (20.0)	10 (33.3)	3 (10.0)
2	Control	4	36	36 (100)	0 (0)	0 (0)	0 (0)
	Li_2CO_3	4	34	13 (38.2)	8 (23.5)	10 (29.5)	3 (8.8)
3	Control	4	34	34 (100)	0 (0)	0 (0)	0 (0)
	Li_2CO_3	4	33	10 (30.3)	8 (24.2)	9 (27.3)	6 (18.2)
